# Selective area epitaxy of ultra-high density InGaN quantum dots by diblock copolymer lithography

**DOI:** 10.1186/1556-276X-6-342

**Published:** 2011-04-15

**Authors:** Guangyu Liu, Hongping Zhao, Jing Zhang, Joo Hyung Park, Luke J Mawst, Nelson Tansu

**Affiliations:** 1 Center for Optical Technologies, Department of Electrical and Computer Engineering, Lehigh University, Bethlehem, PA 18015, USA; 2 Reed Center for Photonics, Department of Electrical and Computer Engineering, University of Wisconsin - Madison, Madison, WI, 53706, USA

## Abstract

Highly uniform InGaN-based quantum dots (QDs) grown on a nanopatterned dielectric layer defined by self-assembled diblock copolymer were performed by metal-organic chemical vapor deposition. The cylindrical-shaped nanopatterns were created on SiN_*x *_layers deposited on a GaN template, which provided the nanopatterning for the epitaxy of ultra-high density QD with uniform size and distribution. Scanning electron microscopy and atomic force microscopy measurements were conducted to investigate the QDs morphology. The InGaN/GaN QDs with density up to 8 × 10^10 ^cm^-2 ^are realized, which represents ultra-high dot density for highly uniform and well-controlled, nitride-based QDs, with QD diameter of approximately 22-25 nm. The photoluminescence (PL) studies indicated the importance of NH_3 _annealing and GaN spacer layer growth for improving the PL intensity of the SiN_*x*_-treated GaN surface, to achieve high optical-quality QDs applicable for photonics devices.

## Introduction

Nitride-based semiconductor devices have tremendous applications in solid-state lighting [1-9], lasers [10-14], photovoltaic [15-17], thermoelectricity [18-20], and terahertz photonics [21,22]. Nitride-based InGaN quantum wells (QWs) are typically employed as active regions in energy-efficient and reliable light-emitting diodes (LEDs) for solid-state lighting. However, the large spontaneous and piezoelectric polarization fields in III-Nitride material lead to a significant charge separation effect [23-35], which in turn results in low internal quantum efficiency of green-emitting nitride-based LEDs, and high threshold current density in nitride lasers. Nonpolar nitrides were employed to remove the polarization field [23]; however, the development of nonpolar InGaN QWs is relatively limited due to high substrate cost and less mature epitaxial techniques. Recent approaches to improve the LED internal quantum efficiency by employing novel InGaN QWs with improved electron-hole wavefunction overlaps have been reported [24-35], as follows: (1) InGaN QW with AlGaN δ-layer [24], (2) staggered InGaN QW [25-30], (3) type-II QW [31], (4) strain-compensated InGaN-AlGaN QW [32,33], (5) InGaN-delta-InN QW [34], and (6) InGaN QW with novel AlInN barrier design [35].

The pursuit of quantum dot (QD)-based active regions for optoelectronic and photovoltaic devices is very important because of the stronger quantum effects in the nanostructures [36-39]. The three-dimensional potential boundaries deeply localize carriers, and thus the overlap of the electron-hole wavefunctions is greatly enhanced. The strain field from the large lattice mismatch of InGaN/GaN is released in three dimensions for QD nanostructures so that the non-radiative recombination centers and defects can significantly be reduced. Besides, QD design enables high In-content InGaN epitaxy, which enlarges the coverage of emission spectrum and enriches the design of QD-based active region. The QDs can be implemented in intermediate-band solar cells [40,41] to greatly enhance the efficiency over the whole solar spectrum.

Two conventional approaches for realizing the QD structure include (1) etching technique and (2) self-assembled epitaxy based on Stranski-Kastranow (S-K) growth mode [42-50]. The approach to obtain QD by etching techniques suffers from surface roughness and significant surface recombination issues. The S-K growth mode has been employed by both molecular beam epitaxy and metal-organic chemical vapor deposition (MOCVD) technique for the epitaxy of nitride-based [42-45] and arsenide-based QDs [46-48].

The MOCVD epitaxy of the self-assembled InGaN QDs emitting in the 510-520-nm region has been reported in reference [44]. The use of the self-assembled growth technique of InGaN QDs led to QDs with circular base diameter of 40 nm and an average height of 4 nm, and the QD's density was measured as 4 × 10^9 ^cm^-2^. The S-K growth mode of InGaN QDs [42-45] resulted in relatively low density range (mid 10^9 ^up to high 10^9 ^cm^-2^), nonuniformity in QD distribution, and the existence of wetting layer. In contrast to InGaN-based QDs, S-K growths of In(Ga)As/GaAs QDs [46-48] have led to high-performance lasers with high QD density (high 10^10 ^cm^-2^) and uniform QD distribution.

Another important obstacle preventing one from fully exploring the radiative and gain properties of the QD structure from S-K growth mode is the inherent presence of the wetting layer [36-38,49,50]. Several recent studies have shown that the strain fields in the wetting layer from the S-K-grown QDs reduces the envelop function overlap and recombination rate in QD's active region [36-38]. The wetting layer also serves as a carrier leakage path because of the coupling of wetting-layer states with localized QD states, which leads to the increase of threshold current in laser devices.

To eliminate of the detrimental wetting layer as well as fully control the formation of QDs, an alternative to achieve the growth of arsenide-based and nitride-based QDs devices by utilizing selective area epitaxy (SAE) [51-57]. The ideal QDs obtained by the SAE approach [52-57], in particular realized by employing diblock copolymer lithography [55-57], have comparable QD density to that of S-K growth mode, but potentially have better device performance because of the removal of the wetting layer and better carrier confinement [55-57]. Previous studies on the SAE of InGaN QDs have been pursued by using electron-beam lithography [58-61], and anodized aluminum oxide (AAO) template [62].

In this study, we present the SAE of ultra-high density and highly uniform InGaN-based QDs on the nano-patterned GaN template realized by diblock copolymer lithography. The diblock copolymer lithography is ideal for device applications due to the adaptability to full wafer scale nanopatterning. All growths were performed by employing MOCVD on GaN templates grown on c-plane sapphire substrates. The distribution and size of QDs are well controlled, and the presence of the wetting layer is eliminated. Our photoluminescence (PL) studies under different template treatments and different growth conditions confirm the effect of SiN_*x *_deposition on the GaN template surface, as well as provide possible solutions to enhance luminescence from the QD samples.

It is to be noted that the use of SAE approach on dielectric nanopatterns defined by diblock copolymer process resulted in the growths of InGaN QDs without wetting layer, which potentially led to the increase in optical matrix element. In addition to the improved matrix element in the QD, the use of dielectric layers also serve as current confinement layer resulting in efficient carrier injection directly into the InGaN QDs arrays. The diblock copolymer lithography approach also leads us to very high-density patterning with excellent uniformity and low cost. In contrast, the use of AAO template leads to relatively non-uniform patterning, while the use of e-beam lithography leads to a high-cost approach.

## Nanopatterning and SAE of InGaN QDs

**Figure 1:**
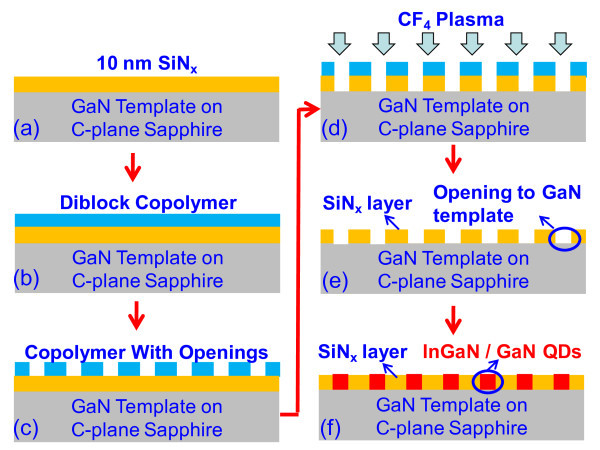
**MOCVD process flow of InGaN/GaN QDs SAE with dielectric patterns defined by the self-assembled diblock copolymer**.

The fabrication process consists of nano-template preparation by diblock copolymer lithography and SAE by MOCVD. Figure 1a-f shows the schematics of the fabrication process flow for the SAE-QDs defined by diblock copolymer approach. The growth of 3-μm GaN template on the c-plane sapphire substrate was carried out by employing MOCVD. The growths of the GaN templates were carried out by employing etch-back and recovery process with 30-nm low-temperature buffer layer [1,7], and the growths of high-temperature GaN layers were carried out at a temperature of 1080°C. Subsequently (Figure 1a), 10 nm SiN_*x *_was deposited on the sample by plasma-enhanced chemical vapor deposition and followed by NH_3 _annealing at a temperature of 800°C for 20 min to increase the adhesion of SiN_*x *_on GaN template.

The sample was then pretreated using PS-r-PMMA brush material followed by the deposition of cylindrical-shaped diblock copolymer PS-b-PMMA (Figure 1b) [55-57]. The brush material is made of random copolymer that would lead to non-preferential affinity to the both blocks of the self-organizing PS-b-PMMA copolymer [55], which enabled the formation of the cylindrical morphology on the diblock copolymer layer during the thermal annealing as a result of the microphase separation. After the UV exposure (λ = 254 nm) and chemical etching by acetic acid, the PMMA block was removed, leaving the PS block to form the patterned copolymer that was used as the polymer stencil (Figure 1c). Subsequently, the sample was made to undergo the reactive ion etching by CF_4 _plasma, and the nanopatterns were transferred from the copolymer layer to the underneath SiN_*x *_layer (Figure 1d). After the removal of the copolymer by O_2 _plasma and wet etching, the SiN_*x *_layer with the nanopatterns could serve as the mask in the following MOCVD process (Figure 1e). The details of the diblock copolymer-processing steps (Figure 1b-e) are described in references [55,56]. The opening region where GaN template was exposed to the metal-organic source would enable the QD growth (Figure 1f). The remaining SiN_*x *_layer can also serve as an insulator between QDs within the active region of a device.

**Figure 2:**
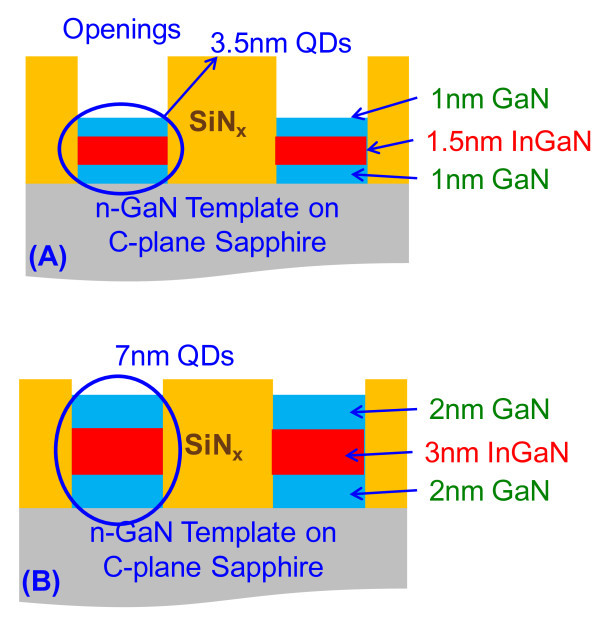
**Schematic of two groups of QD samples with the structures of: (A) 1.5-nm InGaN sandwiched between 1 GaN layers (Sample A); (B) 3 nm InGaN sandwiched between 2-nm GaN layers (Sample B)**.

Both the *n*-GaN template and InGaN QD samples used in this study were grown by a vertical-type VEECO P-75 MOCVD reactor. The growths of the InGaN QD-active region and GaN barrier layers employed triethylgallium, trimethylindium, and ammonia (NH_3_) as gallium, indium, and nitrogen precursors, respectively. The use of trimethlygallium was employed for the growth of *n*-GaN template (*T*_g _= 1080°C). The growth rates for InGaN active and GaN barrier layers in planar region were 3 and 2.4 nm/min, respectively. The growth temperature and growth pressure for the InGaN QDs and GaN barrier layers were kept at 735°C and 200 Torr, respectively. The top GaN barrier layer also serves as the cap layer for the sample, and its similar growth temperature with that of the InGaN QDs leads to minimal dissolution of the In during the barrier layer growth. The V/III molar ratios employed for the growths of the GaN templates, GaN barrier and InGaN active layers were 3900, 34500, and 18500, respectively. Based on growth calibration using XRD measurements, the In-content of the InGaN layer employed in the studies was calibrated as 15%. In our experiments, two sets of structures were investigated as shown in Figure 2, as follows: (1) Sample A consists of 1.5 nm InGaN layer sandwiched between GaN barrier layers each of 1 nm in the opening region with a total thickness designed to be 3.5 nm; and (2) Sample B consists of 3 nm InGaN layer sandwiched between GaN barrier layers of 2 nm each making the total thickness of 7 nm.

## Structural and morphology characterizations

**Figure 3:**
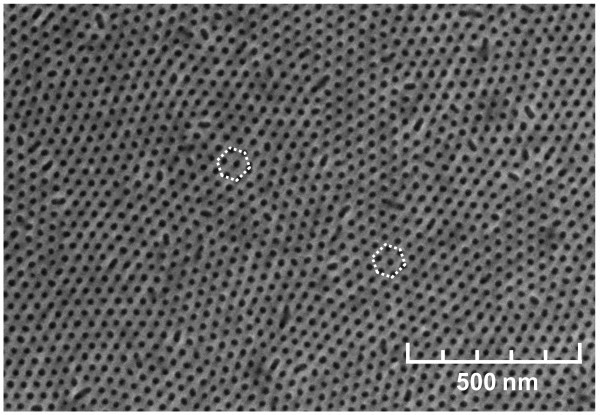
**SEM image of diblock copolymer nanopatterns on SiN**_***x***_** with the hexagonal array of openings after the UV exposure**.

To investigate the surface topographies and QD morphologies, scanning electron microscope (SEM) (Hitachi 4300) and atomic force microscopy (AFM) (Dimension 3000 and Agilent 5500) measurements were performed. Figure 3 shows the SEM image of the copolymer deposited on SiN_*x *_layer after undergoing the UV radiation which would result in nanopore openings, before any active region growth. The SEM images shown in Figure 3 are similar to the processing step described in Figure 1c. The diameter of the holes in the copolymer was measured as approximately 20-25 nm, and the arrangement of the copolymer shows 2-D hexagonal-closed packed structure, although without long-range order between grain boundaries.

**Figure 4:**
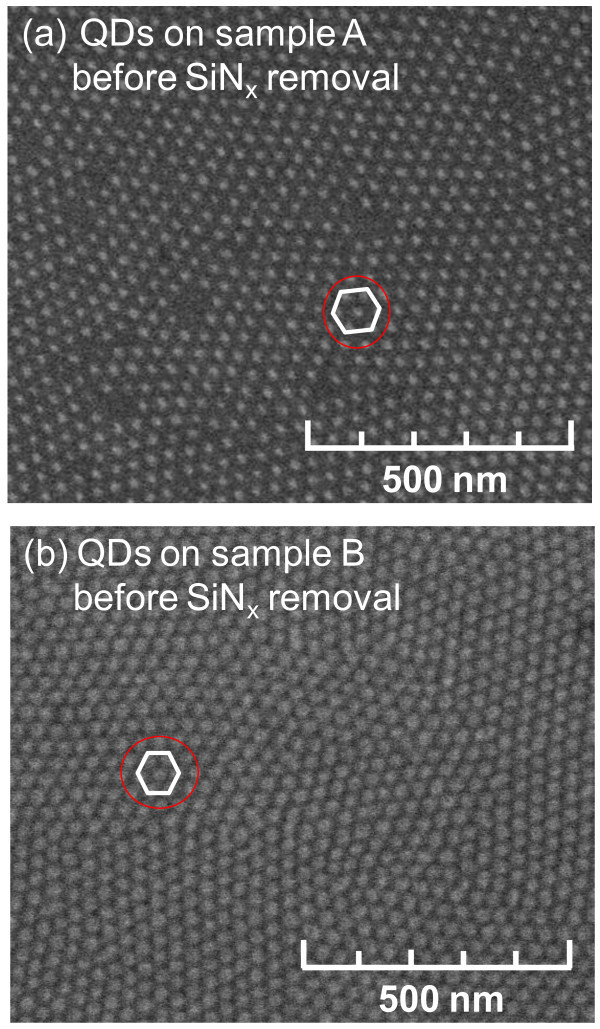
**SEM images of SAE-grown InGaN/GaN QDs with SiN**_***x***_** layer for both samples investigated: (a) sample A; (b) sample B**.

Figure 4a,b shows the SEM images of the samples A and B with InGaN/GaN QDs surrounded by the SiN_*x *_dielectric layer. The SEM measurements demonstrate the successful growth of InGaN/GaN QDs by SAE with the elimination of wetting layer. The hexagonal arrangement of QD arrays on both samples is in good agreement with the arrangement of the openings on copolymer layer as shown in Figure 3.

**Figure 5:**
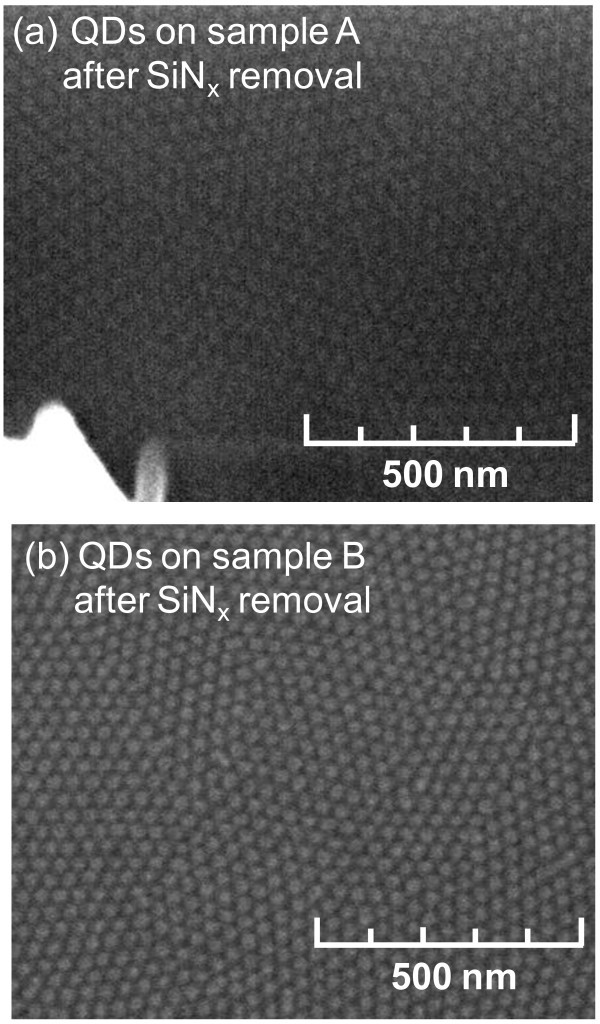
**SEM images of SAE-grown InGaN/GaN QDs after removal of SiNx layer for both samples investigated: (a) sample A; (b) sample B**.

The SEM images of the samples A and B after the removal of SiN_*x *_layer by HF wet etching were shown in Figure 5a,b, respectively. The SEM measurements indicate that the QDs on both the samples were comparable in both the size and distribution with QDs before the elimination of the SiN_*x *_layer. The QD diameters were estimated to be about 22 and 25 nm on the samples A and B, respectively. The QD densities for the samples A and B were measured as 7 × 10 and 8 × 10^10 ^cm^-2^, respectively, which happen to be among the highest QD density reported for InGaN material systems.

Earlier, studies have been carried out to obtain high density nitride-based QDs [63,64]. Krestnikov et al. [63] reported the QD-like behavior in InGaN QW resulting from the In-clustering effect, and the density of the In-riched nanoisland within the QW layer was estimated to be in the range of 10^11^-10^12 ^cm^-2^. The QD-like behavior in InGaN QW from the In-clustering effect resulted in relatively shallow QD/barrier systems. Tu et al. [64] reported the growth of InGaN QDs by employing GaN templates with SiN_*x *_treatment which resulted in template roughening, and this process leads to dot density of near 3 × 10^11 ^cm^-2 ^[64]. However, the use of roughening approach leads to QD distribution with relatively non-uniform size distributions. Thus, the use of SAE approach in growing the InGaN QDs enabled them to grow highly uniform QDs with deep QD/barrier systems (i.e., with GaN or other larger bandgap barrier materials) and very high QD density (approx. 8 × 10^10 ^cm^-2^).

**Figure 6:**
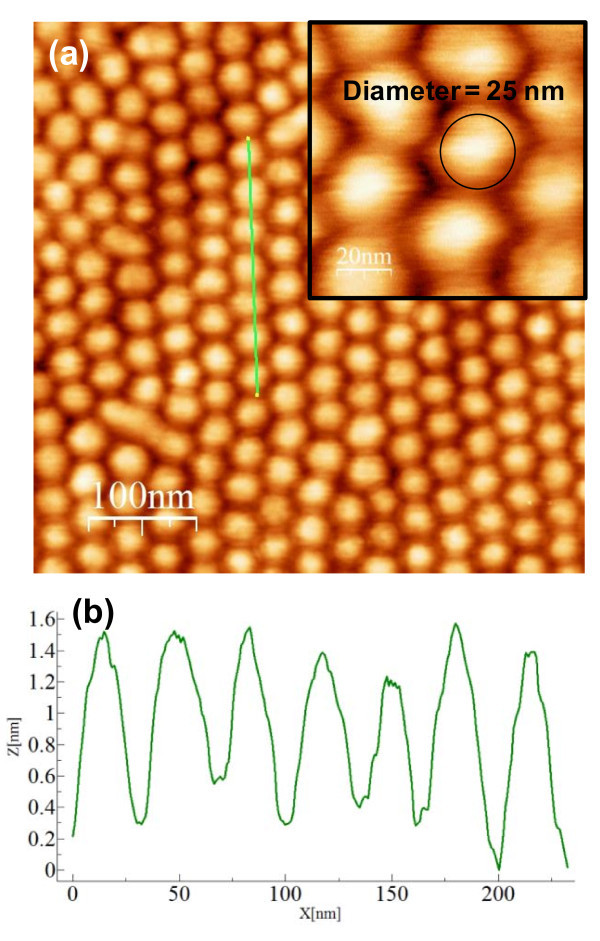
**AFM measurement using Dimension 3000 for SAE-grown InGaN/GaN QDs arrays on sample A after removal of SiN**_***x***_**: (a) AFM scan with the scale of 0.5 μm × 0.5 μm; (b) the corresponding height and size of the cross-sectional profiles**.

AFM measurements on InGaN/GaN QD samples were carried out after the removal of SiN_*x *_layer to provide with direct measurements of QDs morphology. The AFM measurements of the InGaN/GaN (Sample A) were carried out using Dimension 3000, as shown in Figure 6a,b. Figure 6a shows the InGaN/GaN QDs arrays with the scale of 0.5 μm × 0.5 μm, and Figure 6b refers to the height and lateral of the cross-sectional profiles indicated in Figure 6a. The highly uniform QDs were observed from AFM measurements. The dot density was estimated to be 7.5 × 10^10 ^cm^-2 ^with the average height of 1.84 nm and dot diameter of about 25 nm, and these results are in good agreement with those of the nanopatterns employed in the studies. The height and the size of the cross-sectional profiles in Figure 6b indicate that the growth of the dots was well controlled, and the sample exhibits much less variations in dot size, shapes, and distributions compared to those of SK growth mode [44].

**Figure 7:**
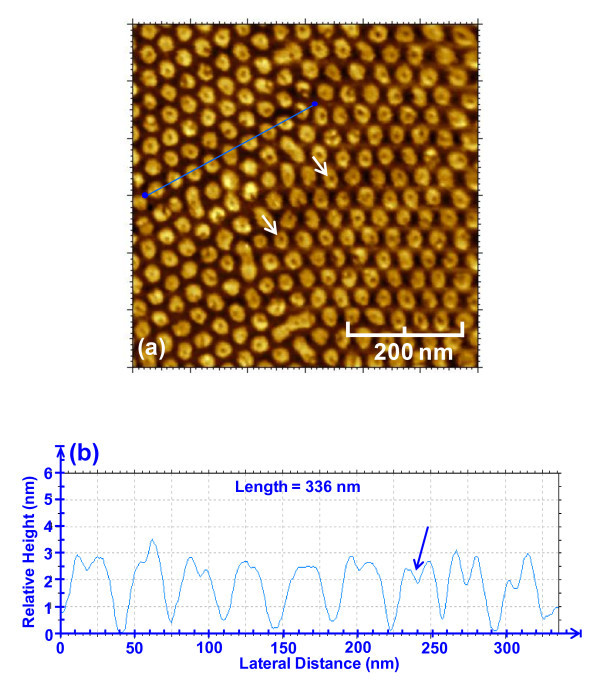
**AFM measurement using Agilent 5500 for SAE-grown InGaN/GaN QDs arrays on sample A after removal of SiN**_***x***_**: (a) AFM scan with the scale of 0.6 μm × 0.6 μm; (b) the corresponding height and size of the cross-sectional profiles**.

For comparison purpose, separate AFM measurements were carried out on sample A by employing Agilent 5500 which consists of higher resolution tip, as shown in Figure 7a,b. Figure 7a shows the AFM image for InGaN/GaN QDs arrays (sample A) with the scale of 0.6 μm × 0.6 μm, and Figure 7b shows the corresponding height and spacing profile for the sample. The QDs were shown to have cylindrical shape, and the QDs density was measured as 7.92 × 10^10 ^cm^-2 ^with average height of 2.5 nm and dot diameter of about 25 nm. The dip-like profile in the QDs could be attributed to different growth rate in the center and outer regions of the QDs, which require further studies to confirm this finding.

**Figure 8:**
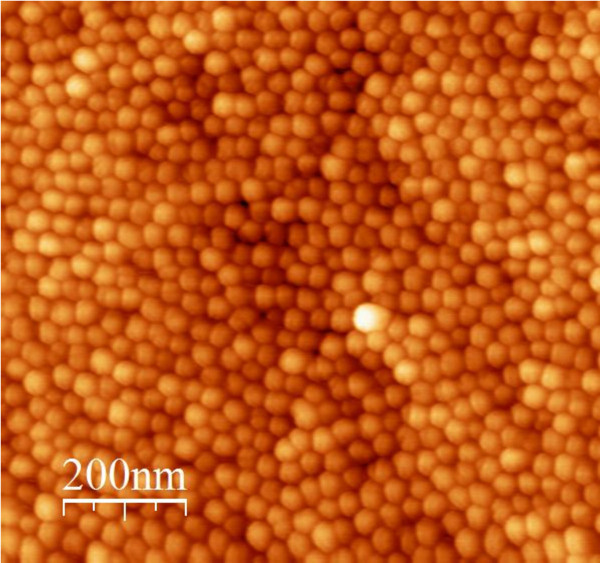
**AFM image of **SAE-grown **InGaN/GaN QDs on samples B measured by Dimension 3000 after removal of SiN**_***x***_** on 1 μm × 1 μm area**.

The AFM image of the InGaN QDs grown on sample B is also shown in Figure 8 with a scale of 1 μm × 1 μm (Dimension 3000). The density of dots on sample B is measured as 8 × 10^10 ^cm^-2 ^with the dot diameter of 25 nm and average height of 4.1 nm. Note that the larger heights in the AFM measurements of the QDs measured in sample B is in agreement with the thicker growths for sample B.

The diameter of the QDs in our experiments was measured in the range of 22-25 nm, which is considered as relatively large QDs. The focus of the current studies is to investigate the various optimizations in the growth and annealing conditions for the development of the SAE technique for InGaN QDs with diblock copolymer lithography, and the current studies are focused on the dimensions of 20-25-nm diameter QDs. In order to obtain stronger quantum effects in the 3D carrier confinement, the QDs are preferably realized with smaller diameters (10-18 nm) [36]. However, the 3D quantum effect in the carrier confinement still exists in the 20-25-nm QD diameter as discussed in the theoretical works in [36]. Future optimization studies on the investigation of SAE InGaN QDs with smaller QDs diameter are of importance for achieving nanostructures with stronger 3D carrier confinement, and the optimization of this approach is required to achieve active regions with high optical quality for device applications.

## PL studies and discussion

The SAE approach enabled the growth of ultra-high density InGaN QDs; however, no strong PL was observed from the InGaN/GaN QD samples. All the PL measurements were carried out by utilization of He-Cd laser with wavelength at 325 nm as the excitation source at room temperature. From our studies, we found that the surface treatment during the SiN_*x *_deposition could be the cause for the defect formation in the GaN surface, which results in poor luminescence from the SAE-grown QD samples. The surface treatment processes for the epitaxy of the QDs include SiN_*x *_deposition, and HF or CF_4 _plasma etching. A series of PL studies on the SAE-grown InGaN QDs were performed to identify and further understand the effects of various treatments on the PL of the samples, which will provide guidance in addressing these issues.

**Figure 9:**
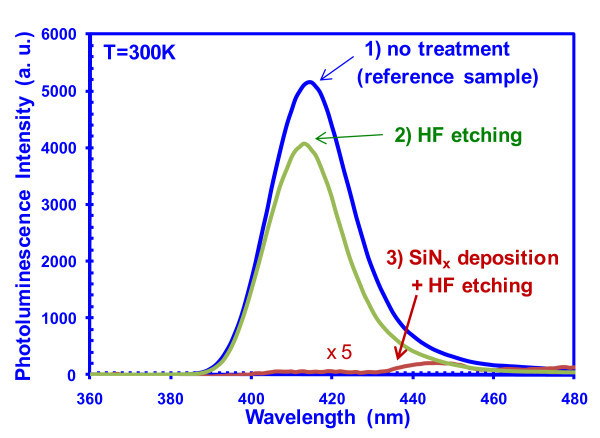
**PL comparison of planar SQW grown on (1) GaN with no surface treatment, (2) GaN with HF wet etching, and (3) GaN with SiN**_***x***_** deposition and HF etching**.

To understand the impact of HF etching on the luminescence properties, the PL spectra comparison of InGaN single-QW samples grown on three different types of GaN template are shown in Figure 9. The active regions in all these samples consist of similar structure; 6 nm GaN barrier followed by 2.5 nm InGaN, and then 10 nm GaN cap layer. The comparison samples include the InGaN single QW grown on three templates as follows: (1) GaN template with no surface treatment (as reference sample), (2) GaN template with HF etching only, and (3) GaN template with SiN_*x *_deposition and HF wet etching. The data indicate that the HF etching does not lead to any detrimental effect on the InGaN QW grown afterward, while the SiN_*x *_deposition process leads to significant detrimental effect on the InGaN QW grown on top of the GaN template as indicated by the significant reduction in the PL intensity.

**Figure 10:**
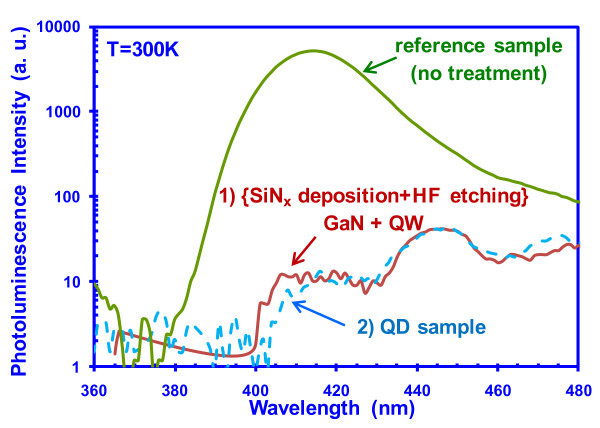
**PL comparison of (1) planar InGaN QW on GaN template that has been treated with SiN**_***x***_** deposition and HF etching, and (2) InGaN QD sample with the same InGaN and GaN layer thickness**.

To confirm the effect of SiN_*x *_deposition on the GaN template surface, PL studies were conducted on two additional types of samples shown in Figure 10, as follows: (1) InGaN QDs grown on nanopatterned GaN template, and (2) planar InGaN QW with the same thickness for InGaN and GaN grown on the GaN templates that had been treated with SiN_*x *_deposition and HF wet etching, i.e., the same process employed to form the dielectric mask for selective QD growth. The spectra for both samples were compared to that of the InGaN QW grown on the GaN template with no surface treatment (reference sample), and very poor PL spectra were observed for both samples grown on the templates that had been treated with SiN_x _deposition and HF wet etching (Figure 10), indicating that the surface modification from the SiN_*x *_deposition on GaN template surface is responsible for the poor luminescence.

**Figure 11:**
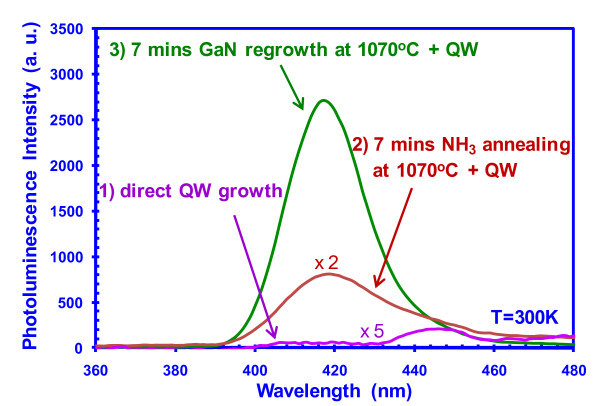
**PL enhancement study of SQW with different growth condition treatments**.

Experiments were carried out to identify possible approaches to address the SiN_*x *_surface treatment issue, as illustrated in Figure 11. Different growth conditions were applied to the GaN templates that had been treated with SiN_*x *_deposition and HF etching, and the same InGaN QWs (6 nm GaN/2.5 nm InGaN/10 nm GaN) were grown afterwards. The PL spectra from InGaN QW directly grown on GaN template undergoing SiN_*x *_deposition and HF etching, without any additional growth treatment are shown in Figure 11 (Direct QW Growth). By annealing the GaN template under NH_3 _environment at 1070°C for 7 min, the single QW grown on the second sample has almost 40 times enhancement in the peak intensity at 420-nm emission. The third sample consisted of a 7-min GaN regrowth at 1070°C before the single-QW growth, and this sample exhibited additional approximately sevenfold improvement in peak intensity as compared to that of the second sample. The series of PL studies indicate that the GaN regrowth and the NH_3 _annealing condition before the QD/QW-active region growth could potentially lead to solutions for addressing the defect generated from the SiN_*x *_deposition on GaN templates. Future studies will involve the application of these procedures to the selective QD growth. Other future approaches by coupling the SAE InGaN QDs with surface plasmon based structures [65,66] will be of great interest for enhancing the radiative efficiency in LED devices.

## Summary

In summary, the selective area growths of InGaN QDs on dielectric patterns defined by the self-assembled diblock copolymer were carried out by MOCVD. The use of selective area approach resulted in ultra-high QD density of approx. 8 × 10^10 ^cm^-2^, which represents the highest among the QD densities reported for highly uniform and well-controlled nitride-based QDs. PL studies of InGaN QDs and the QWs show that GaN spacer regrowth as well as annealing conditions can greatly improve the luminescence from QD samples. The availability of highly uniform and ultra-high density InGaN QDs formed by this approach potentially has significant impacts on developing high-efficiency LEDs for solid-state lighting, low threshold current density-visible diode lasers, and intermediate-band nitride-based solar cells.

## Abbreviations

AAO, anodized aluminum oxide; AFM, atomic force microscopy; LEDs, light-emitting diodes; MOCVD, metal-organic chemical vapor deposition; PL, photoluminescence; QDs, quantum dots; QWs, quantum wells; SAE, selective area epitaxy; SEM, scanning electron microscope.

## Competing interests

The authors declare that they have no competing interests.

## Authors' contributions

NT and LJM initiated, designed, and supervised the experiments carried out in this paper. GY, HZ, and JZ carried out the MOCVD epitaxy, structural and optical characterizations of the InGaN QDs samples grown by the SAE approach. JHP performed the diblock copolymer lithography process as part of the SAE growth experiments. GY, HZ, JZ, NT, LJM analyzed the results. GY, NT, and LJM wrote the manuscript. All authors read and approved the final manuscript.
